# Supplementary material for: Explainable artificial intelligence model to predict acute critical illness from electronic health records
Source: Nat Commun. 2020 Jul 31;11:3852. doi: 10.1038/s41467-020-17431-x (PMC7395744; doi:10.1038/s41467-020-17431-x)
Supplement: Supplementary file 1 — Supplementary Information [file 41467_2020_17431_MOESM1_ESM.pdf]

SUPPLEMENTARY INFORMATION FOR

Explainable artificial intelligence model to predict acute critical illness from electronic health records

Simon Meyer Lauritsen[1,2], Mads Kristensen[1], Mathias Vassard Olsen[3], Morten Skaarup Larsen[3], Katrine Meyer Lauritsen[2], Marianne Johansson Jørgensen[4], Jeppe Lange[2,4], Bo Thiesson[1,5]

[1]Enversion A/S, Fiskerivej 12, 1st floor, 8000 Aarhus C, Denmark; [2]Department of Clinical Medicine, Aarhus University, Palle Juul-Jensens Boulevard 82, 8200 Aarhus N, Denmark; [3] Department of Biomedical Engineering and Informatics, Aalborg University, Niels Jernes Vej 12, 9220 Aalborg Ø, Denmark;[4]Department of Research, Horsens Regional Hospital, Sundvej 30, 8700 Horsens, Denmark; [5]Department of Engineering, Aarhus University, Inge Lehmanns Gade 10, 8000 Aarhus C, Denmark.

**Corresponding author: Simon Meyer Lauritsen (sla@enversion.dk)*

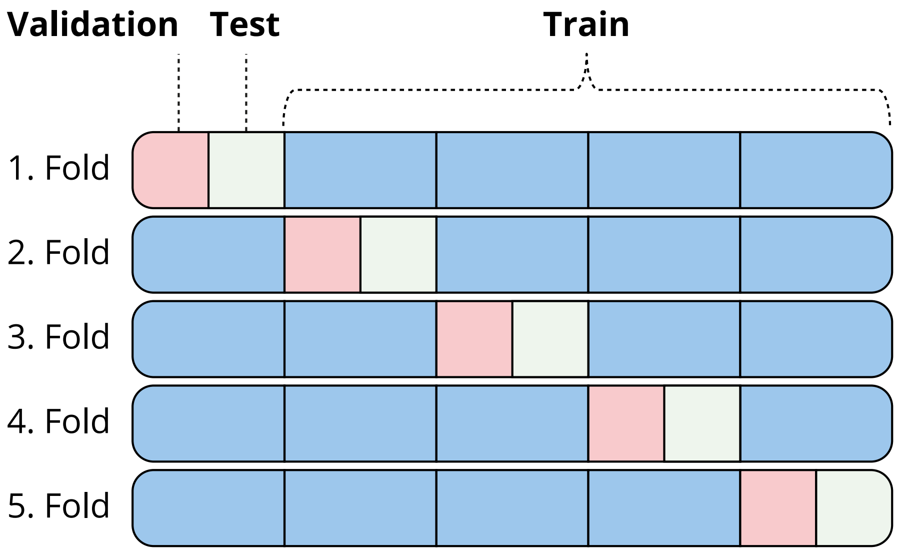

Supplementary Figure 1 | Cross validation scheme. Data were randomly divided into 5 portions of 20% each. For each fold four portions (80 %) was used to fit the xAI-EWS model parameters during training. The remaining 20% was split into two portions of 10% each for validation and test. The validation data were used to perform an unbiased evaluation of a model fit during training, and the test data were used to provide an unbiased evaluation of the final model. For each fold data were shifted such that a new portion was used for testing. All data for a single patient was assigned to either train, validation or test data.

Supplementary Table 1 | The Area Under The Receiver Operating Characteristics (AUROC) curve

		Sepsis				Acute Kidney Injury				Acute Lunge Injury			
Hours before onset	Fold	TCN	SOFA	MEWS	GB-Vital	TCN	SOFA	MEWS	GB-Vital	TCN	SOFA	MEWS	GB-Vital
0	1	0.92	0.83	0.8	0.84	0.88	0.75	0.70	0.76	0.90	0.80	0.85	0.65
0	2	0.95	0.84	0.82	0.83	0.87	0.74	0.68	0.78	0.90	0.80	0.89	0.61
0	3	0.92	0.83	0.82	0.85	0.88	0.74	0.72	0.78	0.93	0.77	0.85	0.63
0	4	0.90	0.83	0.83	0.85	0.9	0.75	0.71	0.76	0.9	0.77	0.81	0.65
0	5	0.93	0.83	0.84	0.83	0.86	0.73	0.70	0.76	0.89	0.82	0.84	0.63
3	1	0.88	0.80	0.78	0.81	0.85	0.74	0.68	0.70	0.85	0.74	0.85	0.63
3	2	0.85	0.80	0.79	0.81	0.84	0.73	0.68	0.72	0.88	0.74	0.78	0.62
3	3	0.85	0.79	0.78	0.81	0.85	0.74	0.70	0.72	0.88	0.72	0.78	0.61
3	4	0.89	0.79	0.78	0.80	0.83	0.75	0.68	0.71	0.85	0.73	0.81	0.55
3	5	0.87	0.8	0.80	0.81	0.86	0.73	0.68	0.75	0.89	0.75	0.85	0.63
6	1	0.86	0.78	0.78	0.78	0.84	0.74	0.70	0.70	0.83	0.73	0.81	0.61
6	2	0.85	0.77	0.77	0.77	0.85	0.72	0.68	0.69	0.82	0.69	0.78	0.63
6	3	0.86	0.79	0.77	0.78	0.85	0.74	0.70	0.69	0.84	0.72	0.78	0.60
6	4	0.86	0.76	0.78	0.80	0.81	0.74	0.69	0.70	0.85	0.75	0.78	0.59
6	5	0.83	0.79	0.77	0.80	0.83	0.73	0.67	0.68	0.88	0.70	0.71	0.63
12	1	0.84	0.77	0.68	0.78	0.85	0.72	0.61	0.68	0.82	0.70	0.74	0.60
12	2	0.83	0.76	0.71	0.77	0.85	0.71	0.69	0.68	0.86	0.76	0.75	0.59
12	3	0.84	0.77	0.71	0.78	0.86	0.72	0.66	0.65	0.84	0.76	0.83	0.61
12	4	0.85	0.77	0.72	0.79	0.85	0.72	0.67	0.68	0.85	0.72	0.77	0.61
12	5	0.84	0.75	0.72	0.79	0.83	0.71	0.61	0.65	0.82	0.70	0.80	0.61
24	1	0.81	0.72	0.71	0.79	0.8	0.66	0.62	0.68	0.85	0.67	0.70	0.60
24	2	0.81	0.72	0.71	0.79	0.77	0.66	0.61	0.60	0.84	0.65	0.71	0.60
24	3	0.80	0.72	0.67	0.77	0.77	0.62	0.60	0.65	0.82	0.70	0.74	0.59
24	4	0.82	0.65	0.65	0.77	0.81	0.67	0.62	0.63	0.85	0.67	0.76	0.58
24	5	0.77	0.71	0.61	0.76	0.79	0.62	0.62	0.60	0.83	0.67	0.75	0.58

TCN: Temporal Convolutional Network, SOFA: Sequential Organ Failure Assessment, MEWS: Early Warning Score, GB-Vital: Gradient Boosting model based on vital sign parameters.

Supplementary Table 2 | Area Under The Precision Recall (AUPRC) Curve

		Sepsis				Acute Kidney Injury				Acute Lunge Injury			
**Hours before onset**	**Fold**	**TCN**	**SOFA**	**MEWS**	**GB-Vital**	**TCN**	**SOFA**	**MEWS**	**GB-Vital**	**TCN**	**SOFA**	**MEWS**	**GB-Vital**
0	1	0.50	0.12	0.12	0.42	0.24	0.11	0.07	0.18	0.24	0.05	0.24	0.05
0	2	0.48	0.11	0.14	0.42	0.21	0.14	0.07	0.20	0.25	0.05	0.12	0.05
0	3	0.38	0.09	0.14	0.42	0.21	0.12	0.06	0.18	0.21	0.04	0.24	0.07
0	4	0.39	0.12	0.13	0.45	0.19	0.12	0.05	0.20	0.25	0.05	0.18	0.05
0	5	0.40	0.11	0.13	0.41	0.23	0.12	0.07	0.17	0.22	0.05	0.19	0.07
3	1	0.12	0.07	0.09	0.10	0.19	0.12	0.08	0.08	0.22	0.05	0.15	0.05
3	2	0.22	0.07	0.06	0.09	0.19	0.11	0.08	0.08	0.23	0.04	0.10	0.04
3	3	0.16	0.09	0.09	0.14	0.23	0.12	0.06	0.07	0.18	0.06	0.20	0.04
3	4	0.18	0.06	0.12	0.11	0.22	0.10	0.05	0.07	0.25	0.05	0.10	0.05
3	5	0.24	0.07	0.08	0.13	0.22	0.10	0.07	0.07	0.22	0.05	0.12	0.05
6	1	0.15	0.06	0.07	0.08	0.21	0.10	0.07	0.03	0.23	0.05	0.15	0.05
6	2	0.12	0.06	0.06	0.05	0.21	0.09	0.05	0.02	0.25	0.03	0.10	0.05
6	3	0.15	0.05	0.12	0.05	0.20	0.11	0.08	0.05	0.19	0.04	0.20	0.07
6	4	0.15	0.05	0.07	0.07	0.19	0.09	0.07	0.02	0.26	0.05	0.09	0.03
6	5	0.13	0.06	0.09	0.05	0.21	0.10	0.06	0.03	0.18	0.05	0.12	0.05
12	1	0.11	0.05	0.04	0.05	0.19	0.08	0.07	0.03	0.20	0.02	0.05	0.05
12	2	0.10	0.05	0.07	0.04	0.19	0.10	0.06	0.02	0.24	0.02	0.04	0.05
12	3	0.12	0.04	0.07	0.05	0.20	0.07	0.06	0.02	0.20	0.05	0.08	0.03
12	4	0.12	0.05	0.07	0.04	0.21	0.08	0.07	0.02	0.21	0.05	0.07	0.03
12	5	0.11	0.09	0.05	0.07	0.19	0.08	0.05	0.03	0.22	0.02	0.07	0.05
24	1	0.09	0.07	0.04	0.07	0.13	0.04	0.02	0.04	0.24	0.02	0.04	0.05
24	2	0.07	0.06	0.05	0.05	0.14	0.05	0.02	0.02	0.22	0.02	0.04	0.04
24	3	0.08	0.04	0.05	0.05	0.13	0.03	0.03	0.02	0.22	0.02	0.05	0.02
24	4	0.08	0.05	0.05	0.08	0.12	0.04	0.04	0.02	0.24	0.02	0.06	0.03
24	5	0.08	0.08	0.03	0.08	0.13	0.04	0.02	0.02	0.23	0.05	0.02	0.02

TCN: Temporal Convolutional Network, SOFA: Sequential Organ Failure Assessment, MEWS: Early Warning Score, GB-Vital: Gradient Boosting model based on vital sign parameters.
